# Neurocognitive Function, Psychosocial Outcome, and Health-Related Quality of Life of the First-Generation Metastatic Melanoma Survivors Treated with Ipilimumab

**DOI:** 10.1155/2020/2192480

**Published:** 2020-07-21

**Authors:** Anne Rogiers, Christophe Leys, Justine Lauwyck, Adrian Schembri, Gil Awada, Julia Katharina Schwarze, Jennifer De Cremer, Peter Theuns, Paul Maruff, Mark De Ridder, Jan L. Bernheim, Bart Neyns

**Affiliations:** ^1^Department of Psychiatry, Centre Hospitalier Universitaire Brugmann, Brussels, Belgium; ^2^Department of Radiotherapy, Universitair Ziekenhuis Brussel, Brussels, Belgium; ^3^Faculty of Psychology, Université Libre de Bruxelles, Brussels, Belgium; ^4^Department of Medical Oncology, Universitair Ziekenhuis Brussel, Brussels, Belgium; ^5^Clinical Science Department, Cogstate Ltd, Melbourne, Australia; ^6^Faculty of Psychology and Educational Sciences, Vrije Universiteit Brussel, Brussels, Belgium; ^7^End-of-Life Care Research Group, Faculty of Medicine, Vrije Universiteit Brussel, Brussels, Belgium

## Abstract

**Purpose:**

To assess neurocognitive function (NCF), psychosocial outcome, health-related quality of life (HRQoL), and long-term effects of immune-related adverse events (irAE) on metastatic melanoma survivors treated with ipilimumab (IPI).

**Methods:**

Melanoma survivors were identified within two study populations (*N* = 104), at a single-center university hospital, and defined as patients who were disease-free for at least 2 years after initiating IPI. Data were collected using 4 patient-reported outcome measures, computerized NCF testing, and a semistructured interview at the start and 1-year follow-up.

**Results:**

Out of 18 eligible survivors, 17 were recruited (5F/12M); median age is 57 years (range 33-86); and median time since initiating IPI was 5.6 years (range 2.1-9.3). The clinical interview revealed that survivors suffered from cancer-related emotional distress such as fear of recurrence (*N* = 8), existential problems (*N* = 2), survivor guilt (*N* = 2), and posttraumatic stress disorder (*N* = 6). The mean EORTC QLQ-C30 Global Score was not significantly different from the European mean of the healthy population. Nine survivors reported anxiety and/or depression (Hospitalization Depression Scale) during the survey. Seven survivors (41%) reported fatigue (Fatigue Severity Scale). Seven patients (41%) had impairment in NCF; only three out of seven survivors had impairment in subjective cognition (Cognitive Failure Questionnaire). Anxiety, depression, fatigue, and neurocognitive symptoms remained stable at the 1-year follow-up. All cases of skin toxicity (*N* = 8), hepatitis (*N* = 1), colitis (*N* = 3), and sarcoidosis (*N* = 1) resolved without impact on HRQoL. Three survivors experienced hypophysitis; all suffered from persistent fatigue and cognitive complaints 5 years after onset. One survivor who experienced a Guillain-Barré-like syndrome suffered from persisting depression, fatigue, and impairment in NCF.

**Conclusion:**

A majority of melanoma survivors treated with IPI continue to suffer from emotional distress and impairment in NCF. Timely detection in order to offer tailored care is imperative, with special attention for survivors with a history of neuroendocrine or neurological irAE. The trial is registered with B.U.N. 143201421920.

## 1. Introduction

Until 2010, no treatment option had improved overall survival (OS) in patients with metastatic melanoma. Since then, effective life-prolonging systemic therapies have been approved of which ipilimumab (IPI), a monoclonal antibody that blocks the cytotoxic T-lymphocyte-associated antigen 4 (CTLA-4) immune checkpoint receptor, was the first. Across studies, treatment with IPI increases the percentage of long-term survival (>3 years) by 10 to 15% [1].

Unfortunately, IPI is also associated with a range of immune-related adverse events (irAE) such as rash, diarrhea, colitis, hepatitis, hypophysitis, and fatigue occurring both during or even after treatment termination [2]. Most of these irAEs are reversible, with the exception of some endocrine and neurological side effects [3]. Given that modulation of immune and endocrine systems also impacts on the normal function of the central nervous system (CNS), immune checkpoint blockade has the potential to give rise to neuropsychiatric symptoms such as depressive mood, anxiety, and impairment in neurocognitive function [4]. Despite this potential, little is known about the long-term effects of immune checkpoint inhibitors (ICI) on neuropsychiatric symptoms in individuals with metastatic melanoma [1].

As many patients with advanced melanoma discontinue their ICI therapy and become long-term cancer survivors, the issue of melanoma survivorship care gains importance [5, 6]. In the field of melanoma, the psychosocial outcome and health-related quality of life (HRQoL) have been studied mainly in individuals with early-stage disease [1]. In these survivors, due to the risk of developing recurrence of melanoma, there is a necessity for continued self-examination, regular dermatological control visits, and reduced sun exposure [7]. Such prevention measures can themselves increase anxiety as well as fear of recurrence, causing denial behavior and leading to decreased self-examination and avoidance of dermatological control visits [8]. The often highly traumatic course of metastatic melanoma may also contribute to greater difficulty in coping when compared to other cancer indications [9].

Studies of psychosocial outcomes in metastatic melanoma survivors are scarce, but all report diminished HRQoL and high levels of distress [10]. Therefore, the aim of this prospective study was to assess the HRQoL and psychosocial and neurocognitive outcomes as well as to document possible sequelae of irAE in survivors of metastatic melanoma treated with IPI. In line with previous findings, the hypothesis was that the first generation of IPI survivors is at high risk of developing emotional distress.

## 2. Methods

This single-center study was undertaken at the Universitair Ziekenhuis Brussel, Brussels, Belgium. Patients were recruited from two prospective studies (ClinicalTrials.gov: NCT02673970 and NCT01302496). This substudy investigating HRQoL and psychosocial and neurocognitive outcomes was approved by the institutional Ethical Committee in 2016 (B.U.N. 143201421920). All patients provided written informed consent.

### 2.1. Study Population

Survivors were recruited by reviewing the databases of the prospective studies. Eligible patients were aged 18 or over, with an unresectable stage IIIC or IV melanoma; survivors were disease-free for at least 2 years following the start of IPI and with no subsequent treatment for their metastatic melanoma.

### 2.2. Procedures

Survivors were contacted by phone and invited to participate. After consenting, they could make an appointment to perform the baseline assessment, defined as *T*_0_, including a clinical interview (60 minutes); completion of patient-reported outcome measures (PROMs) assessing fatigue, HRQoL, anxiety, depression, and impairment in subjective cognitive function (30 minutes); and objective assessment of neurocognitive function (NCF), measured by the Cogstate test battery (40 minutes). All questionnaires were checked for incompleteness, and patients were asked to complete the missing items. Sociodemographic and clinical data were collected from the parental prospective study and a standardized questionnaire.

The baseline assessment took place outside the oncological visits, so as to avoid bias related to distress of the medical control visit. A follow-up assessment was planned one year after baseline, defined as *T*_1_, and comprised the PROMs and the objective assessment of NCF.

Treatment-related toxicity of IPI and adverse events related to adjuvant therapy were documented from the parental prospective studies.

### 2.3. Semistructured Clinical Interview

At the baseline assessment, a semistructured clinical interview was performed by the first author, an experienced psychiatrist in the field of psychooncology. The interview started with the open-ended question on how diagnosis of metastatic melanoma had been announced and what emotions the survivors felt at that moment. Thereafter, a semistructured clinical interview (SCID-IV-CV) based on the Diagnostic and Statistical Manual (DSM-IV-R) was administered to establish a psychiatric diagnosis [11]. Notes of the psychiatric interview were filled in a standardized form. The purpose of this clinical interview was to have additional clinical information to that provided by the PROMs and to assess whether emotional distress was cancer-related. The interview ended with two open-ended questions about the level of fear of cancer recurrence and the degree of uncertainty.

### 2.4. Patient-Reported Outcome Measures

#### 2.4.1. Health-Related Quality of Life

The European Organization for Research and Treatment of Cancer Quality of Life Questionnaire C30 (EORTC QLQ-C30) is a PROM that assesses HRQoL in cancer patients [12]. Taking into account that survivors included in this study had to be disease-free and without cancer treatment for at least 2 years after initiation of IPI, we considered it appropriate to compare mean scores of our survivor population with the European mean of the healthy population. The EORTC QLQ-C30 is composed of 30 items, consisting of 5 functional dimensions (physical, emotional, role, cognitive, and social functioning), 9 symptomatic dimensions (fatigue, nausea/vomiting, pain, dyspnea, insomnia, appetite loss, constipation, diarrhea, and financial impact), which are all scored on a 4-point Likert scale, and one dimension of global HRQoL, measured by 2 items, each scored on a 7-point Likert scale. According to the guidelines, a linear transformation was used to standardize the raw scores from 0 to 100. Differences in scores were considered as clinically relevant according to the guidelines for interpretation of longitudinal HRQoL differences [13]. Thresholds for clinical importance of the EORTC QLQ-C30 were calculated based on the data from Griesinger et al. [14].

#### 2.4.2. Anxiety and Depression

The Hospitalization Anxiety and Depression Scale (HADS) is a 14-item self-report scale, 7 assessing anxiety and 7 depression on a 4-point Likert scale [15]. The HADS has been validated in Dutch and French [16, 17]. A cutoff score of ≥8 has been validated as clinically important to assess emotional distress in oncological settings [18].

#### 2.4.3. Fatigue

The Fatigue Severity Scale (FSS) is a 9-item self-report instrument, on a 7-point Likert scale with scores of ≥4 indicating moderate fatigue [19]. The FSS is validated in Dutch and French [20, 21].

#### 2.4.4. Subjective Cognition

The Cognitive Failure Questionnaire (CFQ) is a 25-item self-report scale, on a 5-point Likert scale [22]. The CFQ measures impairment in subjective cognitive function with scores ≥ 44 indicating impairment in subjective cognition and scores ≥ 55 indicating severe impairment in subjective cognition. The CFQ is validated in Dutch and French [23, 24].

### 2.5. Neurocognitive Function

Neurocognitive function (attention, memory, and executive function) was measured by the objective tests from the Cogstate battery, evaluating processing speed; detection test (DET), attention; identification test (IDN), verbal memory; International Shopping List (ISL) and International Shopping List Delay (ISRL), working memory; One Back test (ONB); and executive function Groton Maze Learning Test (GMLT). The following composite cognitive functions were defined: memory processing speed compound (IDN, DET), memory compound (ISL, ISLR, and ONB), and executive function compound (GMLT, ONB). The Cogstate test battery is validated in the oncological setting [25]. For each survivor, performance on each test was standardized using age-matched normative data [26]. Impairment on a single test was classified when performance was lower than 1 standard deviation below normal age-appropriate mean. Impairment in NCF for an individual was classified when abnormal performance occurred on at least 3 tests of the 7 in the battery according to the guidelines of the International Cognition and Cancer Task Force recommendations [27].

### 2.6. Statistical Analysis

Descriptive statistical analysis was performed using Jamovi (v1.1.7.0) and R3.6.1. Results were considered significant at an alpha level of 0.05 two-sided. Norm-based data of the EORTC-QLQ_C30 from the European Healthy population were compared with the HRQoL outcomes of the study population using one-sample *t*-tests [28].

## 3. Results

### 3.1. Study Population

**Figure 1:**
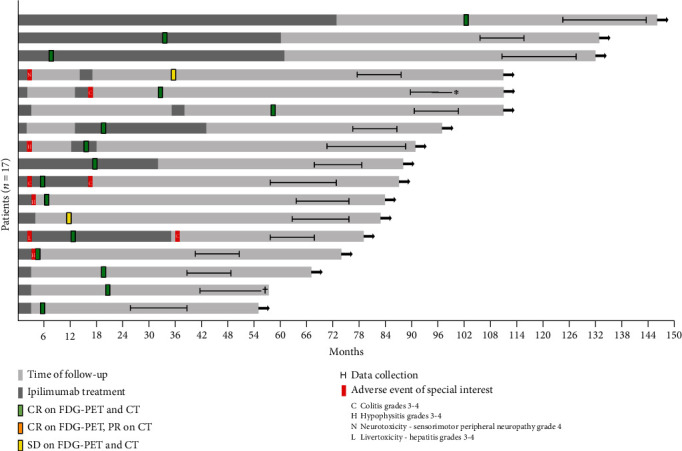
Swimmer plot illustrating duration of IPI administration, date of complete remission (CR), date of stable disease (SD), time of baseline assessment and follow-up, and irAE of special interest. For 2 survivors, no assessment was available at one-year follow-up: one died (†) and one had recurrence of metastatic melanoma (∗).

Of 104 IPI-treated patients considered, 18 survivors (5F/13M), with a median age of 57 years (range 33-86), were eligible, and of these candidates, 17 consented to participate. At baseline assessment (*T*_0_), median time since starting IPI treatment was 5.6 years (range 2.1-9.3) and median time since complete remission or best overall response of metastatic disease was 4 years (range 1.6 to 6.3). Patient characteristics at baseline (*T*_0_) are summarized in Table 1. Fifteen survivors completed the one-year follow-up assessment (*T*_0_): one survivor became ineligible for further assessment due to progression of previously diagnosed prostate cancer, and one survivor had a recurrence of metastatic melanoma. Both patients were in complete remission at study entry (respectively, 1.8 years and 5.2 years). Ipilimumab administration, date of complete remission, data collection, and adverse events of special interest are shown in Figure 1.

### 3.2. Immune-Related Adverse Events

**Figure 2:**
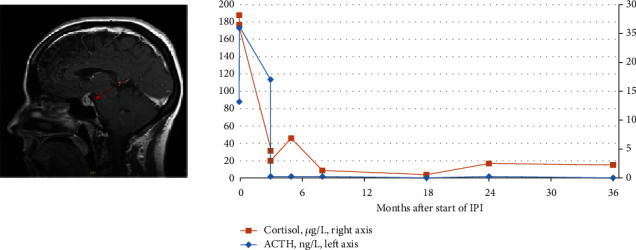
Case illustration. Sagittal section of a gadolinium-enhanced magnetic resonance image from a patient who developed acute symptomatic immune-related hypophysitis leading to an isolated insufficiency of the corticotropic axis. The patient had an Addison crisis three weeks after the fourth administration of IPI (cumulative dose 1080 mg). The MRI image reveals a diffusely swollen and gadolinium-enhanced pituitary gland with a hypointense lesion in the right adenohypophysis and thickening of the stalk. Laboratory findings showed undetectable ACTH levels at the time of diagnosis. The patient was diagnosed with posttraumatic stress disorder (PTSD) related to the symptoms of an Addison crisis with comorbid depressive mood, fatigue, suicidal ideation, psychomotor retardation, and severe subjective neurocognitive symptoms. After one year of psychiatric follow-up, the patient recovered from depression and PTSD; however, he had a recurrence of depression two years later due to fear of recurrence, which again was successfully treated after psychiatric intervention.

Immune-related adverse events (irAE) were retrieved from the databases of the parental prospective studies. Three patients developed hypophysitis, of whom two were subsequently referred for a psychiatric consultation (respectively, 10 and 3 months after the incidence of hypophysitis). The first patient suffered from obsessive-compulsive disorder (OCD) with comorbid depressive disorder and suicidal ideation. The second patient was referred for depressive symptoms, cognitive disturbances, fatigue, and suicidal ideation (Figure 2). The third patient consulted an external psychologist for anxiety and depressive symptoms with suicidal ideation without further follow-up. Only the first patient had a history of a depressive episode, 8 years before diagnosis of melanoma, successfully treated with antidepressants.

All three survivors were treated with a physiological substitution dose of hydrocortisone. The first survivor also remained in need of thyroid and growth hormone substitution and continued to suffer from obsessive compulsive disorder with comorbid depression, severe fatigue, and arthralgia as well as impairment in NCF at baseline line and follow-up assessment. For the second survivor, we found impairment in NCF at baseline and follow-up assessment; depression related to the hypophysitis was resolved at baseline (case illustration Figure 2). The third survivor suffered from persisting cancer-related posttraumatic stress disorder (PTSD) at baseline. The patient-reported outcomes (PROs) revealed that all three suffered from persisting fatigue (FSS *T*_0_ scores = 6; 4 and 4), anxiety (HADS‐A *T*_0_ scores = 10; 9 and 8), depressive symptoms (HADS‐D *T*_0_ scores = 10; 9 and 8), and impairment in subjective cognition (CFQ *T*_0_ scores = 66; 47 and 50) at baseline and at one-year follow-up assessment: fatigue (FSS *T*_1_ scores = 6; 4 and 5), anxiety (HADS‐A *T*_1_ scores = 19; 9 and 8), depressive symptoms (HADS‐D *T*_1_ scores = 14; 4 and 9), and impairment in subjective cognition (CFQ *T*_1_ scores = 72; 45 and 50).

One survivor developed a grade 4 sensorimotor polyneuropathy (Guillain-Barré-like syndrome) that was reported elsewhere [29]. Assessment of PROs reports fatigue (FSS *T*_0_ = 5, *T*_1_ = 4), anxiety and depression (HADS‐A *T*_0_ = 9, *T*_1_ = 12; HADS‐D *T*_0_ = 8, *T*_1_ = 13), and severe impairment in subjective cognition (CFQ at *T*_0_ = 70, *T*_1_ = 79). Assessment of NCF revealed a general impairment in NCF at baseline and at one-year follow-up. According to the SCID-IV-CV, the survivor suffered from a major depressive disorder.

All cases of immune-related skin toxicity (8 patients), hepatitis (1 patient), colitis (3 patients), and sarcoidosis (1 patient), as well as the Guillain-Barré-like syndrome (1 patient) recovered completely.  Table 2 gives an overview of all irAE.

As for non-immune-related long-term toxicity, one survivor suffered from lymphedema related to the resection of the axillar lymph nodes and one had a partial gastrectomy for gastric metastasis.

### 3.3. Semistructured Clinical Interview

The clinical interview revealed that all survivors reported fear of cancer recurrence (FCR) and feelings of uncertainty. Nine pts (53%) reported persisting emotional distress: existential problems (*N* = 2), survivor guilt (*N* = 2), or daily worrying about melanoma recurrence (*N* = 8). Six survivors were classified with cancer-related PTSD (35%) and two survivors with major depressive disorder. The following stressors were reported as life-threatening: rapid disease progression with respiratory distress related to compression of the upper airway by mediastinal lymph node metastases (*N* = 1) and announcement of a diagnosis of inevitably incurable disease with short life expectancy (*N* = 4), and in one survivor, this was related to an acute Addison crisis in the context of a grade 4 hypophysitis (Figure 2). In 8 survivors (47%), no major cancer-related problems were identified.

### 3.4. Patient-Reported Outcomes

#### 3.4.1. Health-Related Quality of Life

At baseline, the mean EORTC QLQ-C30 Global Score was not significantly different from the European mean of the healthy population [28]. Mean scores for cognitive function were significantly lower than their relevant healthy population means, indicating greater impairment. Survivors had no significantly higher symptom level compared to the European mean of healthy subjects [28]. When looking at individual thresholds for clinical importance, we found that 6 survivors (35%) suffered from fatigue and 5 survivors (29%) from impaired emotional function at baseline and at one-year follow-up [14]. Seven patients had no clinically important dysfunction or symptom levels. At the one-year follow-up, one survivor had a clinical improvement of the global HRQoL score (with 17 points), and two worsened (both with 33 points).  Table 3 shows the descriptive statistics of the EORTC QLQ-C30 scores.

#### 3.4.2. Anxiety and Depression

At the baseline and one-year follow-up, a total of 9 survivors (53%) reported clinical levels of anxiety and/or depressive symptoms (HADS ≥ 8). At the baseline, 6 survivors (35%) suffered from anxiety and 7 (41%) from depressive symptoms, whereas 5 (30%) had clinical levels of both anxiety and depressive symptoms. All survivors with clinical levels of anxiety at the baseline had persistent anxiety symptoms at one-year follow-up assessment. Details are illustrated in Table 4.

#### 3.4.3. Fatigue

Seven survivors (41%) suffered from fatigue according to the FSS (≥4) at the baseline, and all of these continued to suffer from fatigue at the one-year follow-up (Table 4).

#### 3.4.4. Subjective Cognition

Seven survivors suffered from impairment in subjective cognition (CFQ ≥ 44) of whom four had scores ≥ 55, indicating severe impairment in subjective cognition (Table 4). Impairment in subjective cognition persisted for all survivors at the one-year follow-up. Six of the seven survivors with impairment in subjective cognition also had persisting high levels of fatigue. The one survivor who developed a brain metastasis (treated with radiotherapy), nine years before baseline assessment, did not have elevated CFQ scores at either baseline or one-year follow-up.

### 3.5. Correlations

Anxiety and depression (HADS), fatigue (FSS), subjective cognition (CFQ), and emotional and cognitive function of HRQoL (EORTC QLQC30) were highly correlated at baseline and at one-year follow-up (Table 5).

### 3.6. Neurocognitive Function

Data from neurocognitive testing was available for 16 survivors; one survivor of 85 years declined assessment for personal reasons. At baseline, seven (44%) survivors had impairment in NCF as defined in the protocol. At the *z* ≤ −1.00 cutoff, 10 patients (62%) were impaired on 2 or more tests. At the one-year follow-up, neurocognitive data were available for 12 survivors of which four (33%) were classified with impairment in NCF; for two survivors with impairment in NCF at baseline, no assessment was available at one-year follow-up. Mean scores are shown in Table 4. Four of the seven survivors with impairment in NCF had no impairment in subjective cognition. The survivor with a history of brain metastasis had no impairment in NCF neither in subjective cognition (CFQ) at baseline and at one-year follow-up. Of the individual tests, only performance on the verbal memory test (ISLT) was correlated significantly with ratings of fatigue (FSS), anxiety and depression (HADS), and subjective cognition (CFQ).

### 3.7. Overview of Results across Measures

The clinical interview revealed that 8 survivors with a good emotional coping did globally very well and had no elevated scores on the HADS, CFQ, and EORTC global and functional scales. Within this group, one survivor reported persisting fatigue, which was confirmed by elevated scores on the FSS and the EORTC fatigue symptom subscale. Of the 7 survivors without any complaints, 4 were classified as showing impairment in NCF.

## 4. Discussion

In this prospective pilot study, we found that a high number (38%) of metastatic melanoma survivors treated with IPI continued to suffer from clinically relevant levels of anxiety, persisting fatigue, and subjective and objective neurocognitive impairment several years after cessation of IPI treatment. These findings from the initial assessment in the survivors were confirmed at a one-year follow-up assessment.

Our preliminary study results showed impairment in NCF in a large proportion (44%) of survivors. In fact, two survivors reported having to stop working due to their cognitive impairment that had persisted after recovery of the disease. The presence of this impairment was confirmed through application of the objective cognitive assessments. The large proportion of survivors with impairment in NCF could be consistent with the study results of Bartels et al., who found that neuronal autoantibodies can provoke neurocognitive impairment, assessed with objective testing in approximately 20% of melanoma patients during treatment (*N* = 157), and that seropositivity of neuronal autoantibodies was also associated with ICI treatment [30]. However, to our knowledge, there are no data available on the influence of cognition, measured objectively, in survivors previously treated with ICI.

Of interest was that impairment in NCF did not accord with impairment in subjective cognition in more than half of the objectively impaired survivors (4/7). Impairment in subjective cognition was associated more strongly with levels of fatigue, anxiety, and depressive symptoms. The high proportion of survivors with impairment in NCF as well as the discrepancy between impairment in subjective cognition and impairment in NCF is also consistent with our previous observations made in short-term metastatic melanoma survivors (in remission since 6 months) treated with the anti-PD1 immune checkpoint inhibitor, pembrolizumab [31].

The occurrence of mood disorders during hypophysitis and neurological irAE might raise the question of whether depression and neurovegetative symptoms should be considered as immune-related adverse events. The current results draw attention to the importance of closely monitoring suicidal ideation as this occurred in all three cases of hypophysitis. Of clinical interest is that in none of these cases, suicidal ideation was reported spontaneously by the patients to their treating oncologist. Our results suggest that the occurrence of neurotoxicity and neuroendocrinological irAEs could potentially have long-term neuropsychiatric and neurovegetative consequences in humans. This suggestion is consistent with the complex interactions between stress, the immune system, the central nervous system, and the tumor microenvironment described previously in preclinical models [32] [33] [34].

The semistructured clinical psychiatric interview revealed that metastatic melanoma survivors suffered from persisting disease-related emotional distress. About half of the survivors (53%) reported fear of cancer recurrence and emotional coping difficulties related to the uncertainty of surviving a potentially lethal disease with a novel treatment of which the long-term outcome remained unknown at the time of the start of this experimental treatment. This is consistent with the findings of Levy et al. describing the burden of uncertainty in patients on active ICI treatment [35]. Posttraumatic stress disorder was present in one-third of the survivors and was related to the moment of receiving a diagnosis of incurable cancer, the traumatic course of disease progression, or the occurrence of irAEs. This is in accord with our previous findings in short-term survivors treated with pembrolizumab as well as with the results of a meta-analysis, where Abbey et al. reported that cancer diagnosis and treatment can induce PTSD [9, 31]. Younger age, completion of treatment, and more advanced disease were found to be associated with a higher risk for developing PTSD [36]. This study population consists of the first generation of survivors, and the high prevalence of PTSD can be better understood when considering that at the time these survivors received their diagnosis, no curative treatment option was available. However, it is of interest that a substantial number of survivors (41%) showed good emotional coping strategies, with no elevated scores on the HADS, CFQ, FSS, or EORTC QLQ-C30 global and functional scales.

According to the EORTC QLQ-C30, and confirming our clinical findings, we found reduced HRQoL in cognitive function. In line with the results on the FSS, HADS, and CFQ, a high number of survivors presented clinical important fatigue symptoms (41%) as well as impaired emotional function (29%) and cognitive function (41%).

Global HRQoL was not different from the normal population. This might be related to the small number of subjects included in this study or to a response shift, which can be explained by an adjustment mechanism to a permanently lower level of global function and emotional wellbeing (due to disease), a well-known phenomenon in HRQoL [37]. In a large cohort study (*N* = 91) comparing IPI survivors with matched controls, Boekhout et al. found decreased physical, role, cognitive, and social functioning as well as increased financial difficulties and fatigue symptoms according to the EORTC QLQ-C30 in IPI survivors [38]. These findings are in line with two recently published surveys, both reporting significant ICI toxicity, psychological concerns such as fear of melanoma recurrence, and impairments in specific HRQoL domains (emotional, physical, role, and social functioning) [38, 39]. Cognitive function was not assessed with an objective NCF test battery.

Limitations of our study are the small sample size, which was anticipated at the start of the study. The number of eligible survivors (17.3%) was in line with the expected number, which was based on the results of an updated report of survival rates of the CA184-014 study and a pooled analysis of 1861 patients, in which the overall survival rate was 18.2% [40, 41]. Due to its small sample size, our study was underpowered to detect all possible long-term effects. It is unlikely that the observations were biased by a high prevalence of preexisting psychoemotional disturbances since such a condition occurred in only one of the studied survivors. In the absence of a control group, normative data were used to assess HRQoL, anxiety, and depression. No causal relationship between IPI treatment and our findings can be concluded as all survivors were heavily pretreated. The strengths of this study are the prospective follow-up design and the combination of PROs with a clinical interview which allows evaluating whether the complaints of emotional distress were disease-related or not. An additional strength is that all survivors achieved long-term remission on IPI for at least 2 years (median time since remission was 4 years) and were off treatment for at least 2 years (median time of stopping therapy was 4 years), which might reduce confounding effects related to pharmacological adverse events or differences between CTLA-4 inhibitors and other ICI.

### 4.1. Clinical Implications

Our study results highlight the need to further address the neurocognitive dysfunction and psychoemotional needs of a rapidly growing new population of melanoma survivors treated with immune checkpoint inhibitors. Special attention to the survivors who suffered from neuroendocrine and CNS irAE seems to be warranted. The treatment outcomes could potentially be improved by offering distress-reducing tailored psychosocial care, which is an additional incentive for further research in the field of immunotherapy, especially in view of the use of ICI in the adjuvant setting as well as in other cancer indications.

### 4.2. Conclusions

Taken together, the current results suggest that in survivors of advanced melanoma, there is continued emotional distress, fatigue, and neurocognitive impairment with an impact on the HRQoL. Timely detection in order to offer tailored care is imperative. Even though this is a relatively small sample size, immunotherapy is developing rapidly, and therefore, we believe that it is important to communicate the robust adverse outcomes of our prospectively studied cohort to the field.

## Figures and Tables

**Table 1:** Baseline characteristics of the study population.

	*N* = 17 (100%)
Sex	
Male	5 (29%)
Median age in years (range)	63.4 (42-85)
Demographics	
Education	
Low (junior high school)	5 (29%)
Intermediate (senior high school)	7 (41%)
High (graduate bachelor or master)	5 (29%)
Work situation	
Work	6 (35%)
Sick leave	3 (18%)
Retired	8 (47%)
Marital status	
Married/cohabitation with partner	12 (71%)
Divorced/separated	2 (12%)
Widowed	2 (12%)
Single/never married	1 (6%)
Children	
Young children (0-21 yrs)	5 (29%)
Adult children (≥21 yrs)	10 (59%)
No children	2 (12%)
Psychiatric history	
Depression	1 (6%)
No psychiatric history	15 (88%)
Psychotropic treatment	
Antidepressant	2 (12%)
Hypnotic benzodiazepine	3 (18%)
ECOG performance status	
0	7 (41%)
1	5 (29%)
2	5 (29%)
Treatment	
Previous treatment before ipilimumab	
Chemotherapy	9 (53%)
BRAF/MEK inhibitors	1 (6%)
Study drug (IFN or TriMixDC-MEL)	7 (41%)
Radiotherapy	9 (53%) non-CNS, 1 (6%) CNS
Surgery	2 (12%)
Therapy during ipilimumab treatment	
Radiotherapy	2 (12%)
Surgery	2 (12%)
Retreatment with ipilimumab after interruption	3 (18%)
Dose of ipilumumab per administration	
3 mg/kg	8 (47%)
10 mg/kg	9 (53%)
Survival	
Number of patients disease-free and without any cancer treatment at baseline^∗^	17 (100%)
Median time since starting ipilimumab at baseline in years (range)	5.6 (2.1-9.3)
Median time since stopping ipilimumab at baseline in years (range)	4.0 (1.9–8.6)
Median time since complete remission or best overall response at baseline in years (range)	4.0 (1.6–8.6)
Number of patients who completed the 1-year follow-up assessment	15 (88%)
Melanoma	
Median time to diagnosis of stages IIIC-IV in years (range)	6.8 (3.4-12.1)
Location of primary tumor	
Head	3 (18%)
Neck	1 (6%)
Trunk	4 (24%)
Upper extremity	2 (12%)
Lower extremity	4 (24%)
Unknown	3 (18%)
AJCC TNM stage 8^th^ edition	
IIIC	2 (12%)
IV-M1a	1 (6%)
IV-M1b	7 (41%)
IV-M1c	6 (35%)
IV-M1d	1 (6%)

^∗^One survivor obtained a complete metabolic response on 18F-FDG/PET and a partial response on CT scan; 1 survivor obtained a stable disease both on 18F-FDG/PET and CT scan; 15 survivors obtained a complete response, defined as the absence of any abnormality on whole-body 18F-FDG PET/CT.

**Table 2:** Overview of immune-related adverse events (IrAEs).

	Grade	Number of IrAE	Intervention
Hypophysitis	3-4	3	Methylprednisolone
Maculopapular rash	1-2	4	Steroid local skin application
Dry skin	2	2	—
Pruritus	1-2	5	Antihistaminic
Diarrhea	1-2	7	—
Colitis	3	4	Methylprednisolone
Hepatitis	3	1	Methylprednisolone
Pneumonitis	3	1	Methylprednisolone
Flu-like syndrome	2	2	—
Guillain-Barré	4	1	Methylprednisolone
Sarcoidosis	2	1	Methylprednisolone
Fatigue	2	4	—

**Table 3:** EORTC QLQ-C30 functional and symptom scales at *T*_0_ and *T*_1_ compared to the pooled European normative data [28].

EORTC QLQ-C30	Mean (SD) *p* value *T*_0_ *N* = 17	Range	Mean (SD) *p* value *T*_1_ *N* = 15	Range	European mean (SD) *N* = 15386
*Function scales*					
Physical	84.3 (15.3) *p* = 0.83	[47-100]	80.9 (16.3) *p* = 0.33	[53-100]	85.1 (18.9)
Role	85.3 (21.2) *p* = 0.85	[33-100]	81.1 (18.7) *p* = 0.52	[50-100]	84.3 (24.6)
Emotional	79.9 (29.0) *p* = 0.43	[0-100]	80.0 (17.5) *p* = 0.22	[50-100]	74.2 (24.7)
Cognitive	72.6 (27.6) *p* = 0.09	[0-100]	64.4 (25.9) *p* = 0.009 *d* = 0.79	[17-100]	84.8 (21.3)
Social	85.3 (21.2) *p* = 0.86	[33-100]	77.8 (25.7) *p* = 0.23	[33-100]	86.2 (24.1)
Global QOL	76.0 (17.4) *p* = 0.03 *d* = 0.47	[50-100]	72.2 (22.9) *p* = 0.33	[42-100]	66.1 (21.7)

*Symptom scales/items*					
Fatigue	27.5 (25.8) *p* = 0.75	[0-78]	21.5 (20.4) *p* = 0.15	[0-67]	29.5 (25.5)
Nausea/vomiting	2.0 (5.5) *p* = 0.01 *d* = 0.59	[0-17]	5.6 (15.0) *p* = 0.93	[0-50]	5.9 (16.0)
Pain	12.7 (16.2) *p* = 0.01 *d* = 0.57	[0-33]	18.9 (23.5) *p* = 0.46	[0-67]	23.5 (27.1)
Dyspnea	13.7 (29.0) *p* = 0.76	[0-100]	15.6 (24.8) *p* = 0.96	[0-67]	15.9 (24.6)
Insomnia	11.8 (16.4) *p* = 0.002 *d* = 0.68	[0-33]	13.3 (21.1) *p* = 0.03 *d* = 0.63	[0-67]	26.6 (30.3)
Appetite loss	7.8 (18.7) *p* = 0.64	[0-67]	15.6 (33.0) *p* = 0.53	[0-100]	10.0 (21.6)
Constipation	5.9 (17.6) *p* = 0.14	[0-67]	9.0 (23.4) *p* = 0.57	[0-67]	12.5 (23.3)
Diarrhea	11.8 (20.2) *p* = 0.65	[0-67]	6.7 (13.8) *p* = 0.45	[0-33]	9.5 (20.9)
Financial difficulties	13.7 (33.5) *p* = 0.71	[0-100]	13.4 (24.7) *p* = 0.67	[0-67]	10.6 (23.6)

Bilateral one sample *t*-tests have been conducted to compare the means with the normative data of the pooled European normative data. *d* = Cohen′s *d* effect size (0.2 = small effect, 0.5 = medium effect, and 0.8 = large effect). Functional scales: a lower score compared to the European mean indicates a worse functioning. Symptom scales: a higher score compared to the European mean indicates more symptoms.

**Table 4:** Descriptive statistics of anxiety, depression, fatigue, subjective and objective cognitive impairment, and number of patients impaired.

	*T*_0_ *N* = 17	*T*_1_ *N* = 15
Anxiety and depression		
HADS anxiety (mean, SD)	5.8 (6.0)	6.4 (6.4)
HADS depression (mean, SD)	5.5 (3.4)	5.4 (4.7)
Number of patients (%) with elevated A scores (≥8)	6 (35%)	6 (40%)
Number of patients (%) with elevated D scores (≥8)	7 (41%)	4 (27%)
Number of patients (%) with elevated A and D scores (≥8)	5 (30%)	4 (27%)
Fatigue		
FSS (mean, SD)	3.0 (1.8)	3.1 (1.5)
Number of patients (%) with elevated scores (≥4)	7 (41%)	7 (47%)
Subjective cognition		
CFQ (mean, SD)	39.8 (23.1)	40.7 (23.7)
Number of patients (%) with elevated scores (≥44)	7 (41%)	7 (47%)

Cognitive computerized test results for processing speed, memory, and executive function composites	*T*_0_ *N* = 16^^^^ Mean (SD)	*T*_1_ *N* = 12^^^^ Mean (SD)
Processing speed composite	-0.67 (1.00)	-1.20 (0.98)
Memory composite	-0.23 (0.97)	0.03 (0.83)
Executive function composite	-0.31 (0.79)	-0.30 (0.90)
Number (%) of patients impaired on objective computerized testing^^^	7 (44%)	4 (33%)

^^^Cognitive impairment was defined when abnormal performance (*z* ≤ -1.00) occurred on at least 3 tests of the 7 in the battery. ^^^^For, respectively, 1 at T0 and 4 patients at 1-year follow-up, no assessment was available.

**Table:** (a) Descriptive statistics and correlations between main variables at baseline (*T*_0_)

	*M* (SD)	FSS^1^	Anx^2^	Dep^3^	CFQ^4^	Glob^5^	CF^6^	EF^7^	PF^8^	RF^9^	SF^10^	FA^11^
FSS^1^	3.0 (1.8)		0.71^∗∗^	0.49^∗^	0.76^∗∗∗^	-0.41	-0.18	-0.66^∗∗^	-0.25	-0.26	-0.34	0.75^∗∗∗^
Anx^2^	5.8 (6.0)			0.30	0.78^∗∗∗^	-0.48^m^	-0.23	-0.84^∗∗∗^	-0.29	-0.26	-0.51^∗^	0.67^∗∗^
Dep^3^	5.5 (3.4)				0.24	-0.24	-0.31	-0.37	-0.28	-54^∗^	-0.09	0.42
CFQ^4^	39.8 (23.1)					-0.43^m^	-0.51^∗^	-73^∗∗∗^	-0.24	-0.10	-0.34	0.78^∗∗∗^

**Table:** (b) Descriptive statistics and correlations between main variables at one-year follow-up (*T*_1_)

	*M* (SD)	FSS^1^	Anx^2^	Dep^3^	CFQ^4^	Glob^5^	CF^6^	EF^7^	PF^8^	RF^9^	SF^10^	FA^11^
FSS^1^	2.9 (1.5)		0.60^∗∗^	0.46^m^	0.73^∗∗^	-0.04	-0.52^∗^	-0.49^m^	0.04	-0.31	-0.29	0.32
Anx^2^	6.1 (6.1)			0.81^∗∗∗^	0.73^∗∗∗^	-0.32	-0.83^∗∗∗^	-0.86^∗∗∗^	0.10	-0.46^m^	-0.56^∗^	0.27
Dep^3^	4.9 (4.6)				0.66^∗∗^	-0.68^∗∗^	-0.91^∗∗∗^	-0.77^∗∗∗^	-0.37	-0.73^∗∗^	-0.65^∗∗^	0.3
CFQ^4^	40.5 (22.2)					-0.16	-74^∗∗^	-0.71^∗∗^	0.10	-0.33	-0.39	0.27

Note: m = marginally significant. ^∗^*p* < 0.05; ^∗∗^*p* < 0.01. diagonal = variances. ^1^Fatigue Severity Scale; ^2^HADS Anxiety; ^3^HADS Depression; ^4^Cognitive Failure Questionnaire; ^5^global HRQoL EORTC QLQ-C30; ^6^Cognitive Function EORTC QLQ-C30; ^7^Emotional Function EORTC QLQ-C30; ^8^Physical Function EORTC QLQ-C30; ^9^Role Function EORTC QLQ-C30; ^10^Social Function EORTC QLQ-C30; ^11^Symptom Scale EORTC QLQ-C30.

## Data Availability

Data are available upon request.

## References

[1] Rogiers A., Boekhout A., Schwarze J. K., Awada G., Blank C. U., Neyns B. (2019). Long-term survival, quality of life, and psychosocial outcomes in advanced melanoma patients treated with immune checkpoint inhibitors. *Journal of Oncology*.

[2] Postow M. A., Hellmann M. D. (2018). Adverse events associated with immune checkpoint blockade. *The New England Journal of Medicine*.

[3] Johnson D. B., Friedman D. L., Berry E. (2015). Survivorship in immune therapy: assessing chronic immune toxicities, health outcomes, and functional status among long-term ipilimumab survivors at a single referral center. *Cancer Immunology Research*.

[4] Kovacs D., Kovacs P., Eszlari N., Gonda X., Juhasz G. (2016). Psychological side effects of immune therapies: symptoms and pathomechanism. *Current Opinion in Pharmacology*.

[5] Langbaum T., Smith T. J. (2019). Time to study metastatic-cancer survivorship. *The New England Journal of Medicine*.

[6] Cheung W. Y., Bayliss M. S., White M. K. (2018). Humanistic burden of disease for patients with advanced melanoma in Canada. *Supportive Care in Cancer*.

[7] McLoone J., Watts K., Menzies S., Meiser B., Butow P., Kasparian N. (2012). When the risks are high: psychological adjustment among melanoma survivors at high risk of developing new primary disease. *Qualitative Health Research*.

[8] Palesh O., Aldridge-Gerry A., Bugos K. (2014). Health behaviors and needs of melanoma survivors. *Supportive Care in Cancer*.

[9] Abbey G., Thompson S. B. N., Hickish T., Heathcote D. (2015). A meta-analysis of prevalence rates and moderating factors for cancer-related post-traumatic stress disorder. *Psycho-Oncology*.

[10] Dunn J., Watson M., Aitken J. F., Hyde M. K. (2017). Systematic review of psychosocial outcomes for patients with advanced melanoma. *Psycho-Oncology*.

[11] Ventura J., Liberman R. P., Green M. F., Shaner A., Mintz J. (1998). Training and quality assurance with the Structured Clinical Interview for DSM-IV (SCID-I/P). *Psychiatry Research*.

[12] Aaronson N. K., Ahmedzai S., Bergman B. (1993). The European Organization for Research and Treatment of Cancer QLQ-C30: a quality-of-life instrument for use in international clinical trials in oncology. *Journal of the National Cancer Institute*.

[13] Cocks K., King M. T., Velikova G. (2012). Evidence-based guidelines for interpreting change scores for the European Organisation for the Research and Treatment of Cancer Quality of Life Questionnaire Core 30. *European Journal of Cancer*.

[14] Giesinger J. M., Loth F. L. C., Aaronson N. K. (2020). Thresholds for clinical importance were established to improve interpretation of the EORTC QLQ-C30 in clinical practice and research. *Journal of Clinical Epidemiology*.

[15] Zigmond A. S., Snaith R. P. (1983). The hospital anxiety and depression scale. *Acta Psychiatrica Scandinavica*.

[16] Spinhoven P., Ormel J., Sloekers P. P. A., Kempen G. I. J. M., speckens a.e.m., hemert A. M. V. (1997). A validation study of the Hospital Anxiety and Depression Scale (HADS) in different groups of Dutch subjects. *Psychological Medicine*.

[17] Bocerean C., Dupret E. (2014). A validation study of the Hospital Anxiety and Depression Scale (HADS) in a large sample of French employees. *BMC Psychiatry*.

[18] Castelli L., Binaschi L., Caldera P., Mussa A., Torta R. (2011). Fast screening of depression in cancer patients: the effectiveness of the HADS. *European Journal of Cancer Care*.

[19] Krupp L. B., LaRocca N. G., Muir-Nash J., Steinberg A. D. (1989). The fatigue severity scale. Application to patients with multiple sclerosis and systemic lupus erythematosus. *Archives of Neurology*.

[20] Debouverie M., Pittion S., Guillemin F., Vespignani H. (2002). Fatigue scales used in multiple sclerosis. *Revista de Neurología*.

[21] Rietberg M. B., van Wegen E. E. H., Kwakkel G. (2010). Measuring fatigue in patients with multiple sclerosis: reproducibility, responsiveness and concurrent validity of three Dutch self-report questionnaires. *Disability and Rehabilitation*.

[22] Broadbent D. E., Cooper P. F., FitzGerald P., Parkes K. R. (1982). The Cognitive Failures Questionnaire (CFQ) and its correlates. *The British Journal of Clinical Psychology*.

[23] Ponds R. W. H. M., Van Boxtel M., Jolles J. (2006). De Cognitive Failure Questionnaire als maat voor subjectief cognitief functioneren. *Tijdschrift voor neuropsychologie*.

[24] Carré (2014). Everyday life psychopathologie: validation of a new cognitve failure's questionnaire. *Psychologie française*.

[25] Vardy J., Wong K., Yi Q. L. (2006). Assessing cognitive function in cancer patients. *Supportive Care in Cancer*.

[26] Cogstate L. (2017). *Cogstate pediatric and adult normative data*.

[27] Wefel J. S., Vardy J., Ahles T., Schagen S. B. (2011). International Cognition and Cancer Task Force recommendations to harmonise studies of cognitive function in patients with cancer. *The Lancet Oncology*.

[28] Nolte S., Liegl G., Petersen M. A. (2019). General population normative data for the EORTC QLQ-C30 health-related quality of life questionnaire based on 15,386 persons across 13 European countries, Canada and the Unites States. *European Journal of Cancer*.

[29] Wilgenhof S., Neyns B. (2011). Anti-CTLA-4 antibody-induced Guillain–Barré syndrome in a melanoma patient. *Annals of Oncology*.

[30] Bartels F., Stronisch T., Farmer K., Rentzsch K., Kiecker F., Finke C. (2019). Neuronal autoantibodies associated with cognitive impairment in melanoma patients. *Annals of Oncology*.

[31] Rogiers A., Leys C., De Cremer J. (2020). Health-related quality of life, emotional burden, and neurocognitive function in the first generation of metastatic melanoma survivors treated with pembrolizumab: a longitudinal pilot study. *Supportive Care in Cancer*.

[32] McGinnis G. J., Raber J. (2017). CNS side effects of immune checkpoint inhibitors: preclinical models, genetics and multimodality therapy. *Immunotherapy*.

[33] Miyajima M., Zhang B., Sugiura Y. (2017). Metabolic shift induced by systemic activation of T cells in PD-1-deficient mice perturbs brain monoamines and emotional behavior. *Nature Immunology*.

[34] Eng J. W.-L., Kokolus K. M., Reed C. B., Hylander B. L., Ma W. W., Repasky E. A. (2014). A nervous tumor microenvironment: the impact of adrenergic stress on cancer cells, immunosuppression, and immunotherapeutic response. *Cancer Immunology, Immunotherapy*.

[35] Levy D., Dhillon H. M., Lomax A. (2019). Certainty within uncertainty: a qualitative study of the experience of metastatic melanoma patients undergoing pembrolizumab immunotherapy. *Supportive Care in Cancer*.

[36] Hahn E. E., Hays R. D., Kahn K. L., Litwin M. S., Ganz P. A. (2015). Post-traumatic stress symptoms in cancer survivors: relationship to the impact of cancer scale and other associated risk factors. *Psycho-Oncology*.

[37] Hinz A., Finck Barboza C., Zenger M., Singer S., Schwalenberg T., Stolzenburg J. U. (2011). Response shift in the assessment of anxiety, depression and perceived health in urologic cancer patients: an individual perspective. *European Journal of Cancer Care*.

[38] Boekhout A. H., Rogiers A., Jóźwiak K. (2019). Health-related quality of life of advanced melanoma survivors treated with CTLA-4 immune checkpoint inhibition: A matched cohort study. *Annals of Oncology*.

[39] O’Reilly A., Hughes P., Mann J. (2020). An immunotherapy survivor population: health-related quality of life and toxicity in patients with metastatic melanoma treated with immune checkpoint inhibitors. *Supportive Care in Cancer*.

[40] Schadendorf D., Hodi F. S., Robert C. (2015). Pooled analysis of long-term survival data from phase II and phase III trials of ipilimumab in unresectable or metastatic melanoma. *Journal of Clinical Oncology*.

[41] Maio M., Grob J. J., Aamdal S. (2015). Five-year survival rates for treatment-naive patients with advanced melanoma who received ipilimumab plus dacarbazine in a phase III trial. *Journal of Clinical Oncology*.

